# Acute Exposure to Microplastics Induced Changes in Behavior and Inflammation in Young and Old Mice

**DOI:** 10.3390/ijms241512308

**Published:** 2023-08-01

**Authors:** Lauren Gaspar, Sydney Bartman, Giuseppe Coppotelli, Jaime M. Ross

**Affiliations:** 1 George and Anne Ryan Institute for Neuroscience, University of Rhode Island, Kingston, RI 02881, USA; 2 Department of Biomedical and Pharmaceutical Sciences, College of Pharmacy, University of Rhode Island, Kingston, RI 02881, USA

**Keywords:** microplastics, aging, mice, behavior, cognition, inflammation

## Abstract

Environmental pollutants have become quite ubiquitous over the past two centuries; of those, plastics, and in particular, microplastics (<5 mm), are among the most pervasive pollutants. Microplastics (MPs) have found their way into the air, water system, and food chain and are either purposely produced or are derived from the breakdown of larger plastic materials. Despite the societal advancements that plastics have allowed, the mismanagement of plastic waste has become a pressing global issue. Pioneering studies on MPs toxicity have shown that exposure to MPs induces oxidative stress, inflammation, and decreased cell viability in marine organisms. Current research suggests that these MPs are transported throughout the environment and can accumulate in human tissues; however, research on the health effects of MPs, especially in mammals, is still very limited. This has led our group to explore the biological and cognitive consequences of exposure to MPs in a rodent model. Following a three-week exposure to water treated with fluorescently-labeled pristine polystyrene MPs, young and old C57BL/6J mice were assessed using behavioral assays, such as open-field and light–dark preference, followed by tissue analyses using fluorescent immunohistochemistry, Western blot, and qPCR. Data from these assays suggest that short-term exposure to MPs induces both behavioral changes as well as alterations in immune markers in liver and brain tissues. Additionally, we noted that these changes differed depending on age, indicating a possible age-dependent effect. These findings suggest the need for further research to better understand the mechanisms by which microplastics may induce physiological and cognitive changes.

## 1. Introduction

Plastics, which are durable, low-cost, and rapidly produced [1], have contributed to some of the greatest recent advancements in society, including modern technology and medical advancements, such as single-use syringes and modern prosthetics [2,3]. These breakthroughs, in combination with many other daily uses for plastics, have led to an almost exponential increase in global plastic production over the past century that has now surpassed 400 million tons per year, with a projected increase to over 1 billion tons within the next ~30 years [4]. This booming plastic production, however, has also led to a significant pollution problem as up to ~70% of the world’s plastic ends up in landfills or is mismanaged in the environment [4]. Plastics in the environment have been shown to leach harmful chemicals [5,6], can be ingested by marine organisms [7], and potentially serve as a transport method for invasive species such as viruses.

In addition to these direct harmful impacts, plastics exposed to environmental factors, such as UV radiation, oxidation, and physical abrasion, have been shown to result in the formation of microplastics (MPs) [8]. Microplastics (MPs) are also purposely produced for use in paints, detergents, and personal care products, such as toothpaste, sunscreen, and cosmetics [9,10]. Microplastics are defined as plastic particles less than 5 mm in diameter and have been shown to have adverse health effects in vitro [11,12] and in vivo [8,13]. Humans are exposed to MPs through the consumption of water, seafood, consumer products (clothes, toothpaste, salt, sugar, honey, beer, anything stored in plastic bottles, plastic wrap, or cans/cartons lined with plastic), and via inhalation from textiles, synthetic rubber tires, and plastic covers [14,15,16]. MPs have been reported to induce oxidative stress [17,18,19], upregulate pro-inflammatory cytokines [20,21,22], decrease cell viability [21,23], and alter energy metabolism [24,25,26], amongst other negative outcomes.

More recently, MPs have been detected in human feces [27,28], cirrhotic liver tissues [29], lungs [30,31], blood [32,33], and even breastmilk [34]. With these discoveries and given that a large portion of research into MPs is still performed in marine models, it has become increasingly important to understand the health outcomes of microplastics exposure in mammals. Currently, there are limited studies that address the potential adverse effects of exposure to MPs on brain health in mammals and even fewer studies that consider age as an additional factor that may impact the outcome of exposure to MPs. Thus, we proposed to investigate the effect that exposure to MPs has on young and old C57BL/6J mice, focusing on neurobehavioral effects, inflammatory response, as well as translocation and accumulation of MPs in tissues, including the brain.

## 2. Results

### 2.1. Cell Viability and Cellular Uptake

**Figure 1:**
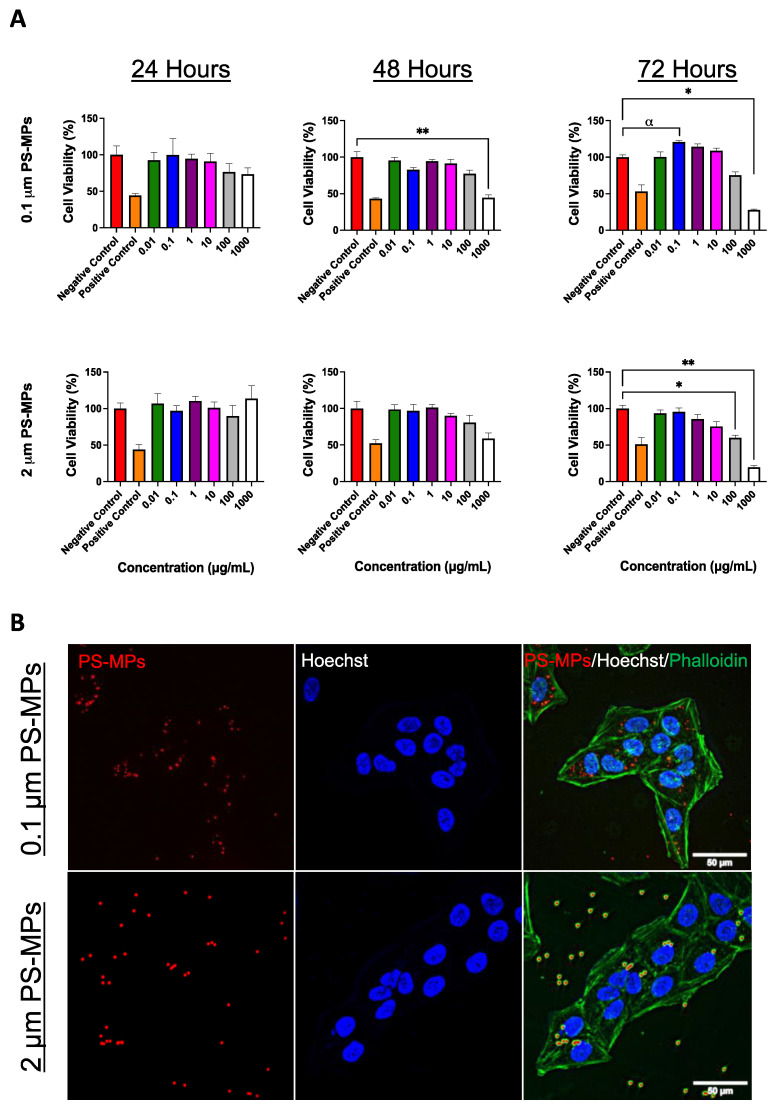
MTT assay and localization of PS-MPs in cells. (**A**) MTT assay to assess cell viability in U-2 OS cells following exposure to PS-MPs. Data shown for 0.1 and 2 μm PS-MPs at concentrations ranging from 0.01 to 1000 μg/mL and exposure times of 24, 48, and 72 h. Significances were determined by one-way ANOVA with post hoc analysis and * *p* < 0.05, ** *p* < 0.01, and α *p* < 0.10 as trending. Data shown are from two or more experiments. (**B**) Representative images of 0.1 and 2 μm PS-MPs (left panel, red) localized in U-2 OS cells counterstained with Hoechst (middle panel, blue) and phalloidin (right panel, green). Scale bar = 50 μm.

In order to study the effect that MPs have in vivo, we first tested cellular uptake and viability in vitro after exposure to commercially available pristine fluorescent polystyrene particles (PS-MPs). U-2 OS cells were cultured and subsequently treated with 0.1 and 2 μm PS-MPs at concentrations ranging from 0.01 to 1000 μg/mL. Following exposure times of 24, 48, and 72 h, cell viability was assessed via MTT assay. After 48 and 72 h, PS-MPs of both sizes induced a dramatic decrease in cell viability, which became more exaggerated with increased concentrations (Figure 1A). Microscopic analysis revealed internalization of PS-MPs in U-2 OS cells as early as after 24 h of exposure (Figure 1B).

### 2.2. In Vivo Exposure and Behavioral Studies

**Figure 2:**
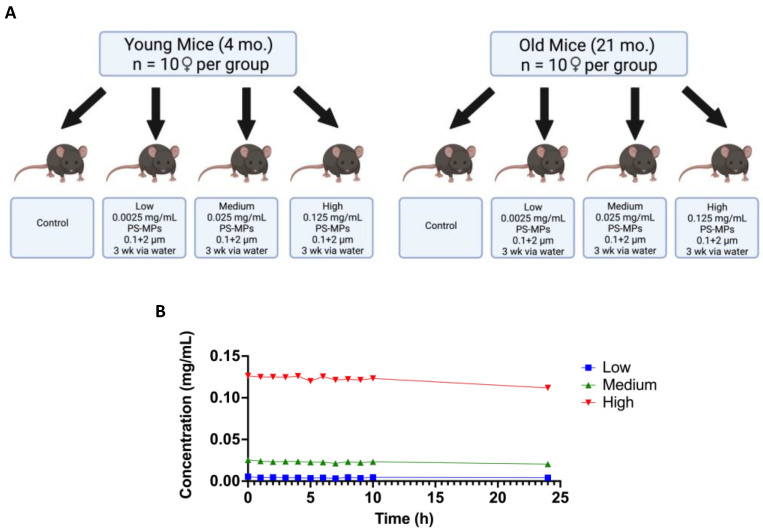
In vivo study design and PS-MPs delivery system concentration curve. (**A**) In vivo study design for short-term (3 weeks) exposure to PS-MPs in young (4 month-old) and old (21 month-old) female C57BL/6J mice (*n* = 10 per group). Schematic was created with BioRender.com (accessed on 2 December 2021). (**B**) Concentration of each dose (low, medium, high) of PS-MPs was measured hourly for 10 h without resuspension and again at 24 h without any resuspension.

Next, we tested the effect of exposure to MPs in young (4-month-old, *n* = 40) and old (21-month-old, *n* = 40) C57BL/6J female mice. Animals were divided into four exposure groups (*n* = 10 per group): normal drinking water (control), 0.0025 mg/mL (low-dose), 0.025 mg/mL (medium-dose), and 0.125 mg/mL (high-dose) water treated with a 1:1 mixture of 0.1 and 2 μm PS-MPs (Figure 2A). All mice were exposed to the appropriate dose of PS-MPs for 3 weeks via water delivery. To ensure that sedimentation of the PS-MPs would not drastically alter concentration throughout the day, each dosage was tested hourly for 10 h, followed by a measurement at 24 h. No significant changes in concentration were found throughout the 24 h period (Figure 2B). We also monitored water consumption and body weights and did not find any alterations in either parameter (Supplementary Figure S1A–D).

**Figure 3:**
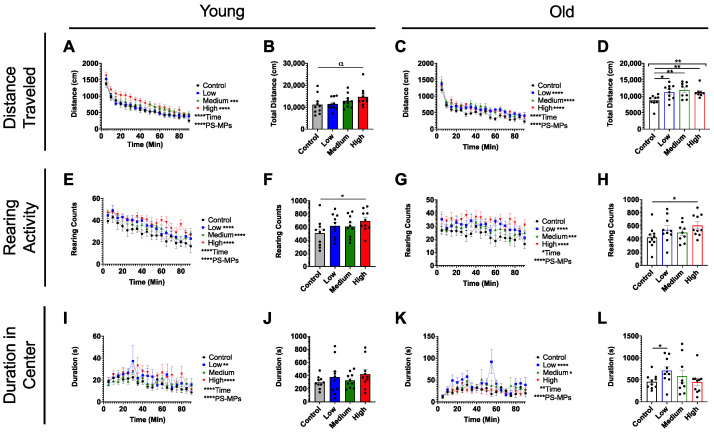
Effects of exposure to PS-MPs on locomotion in young and old mice. Spontaneous locomotor activity of 4- and 21-month-old female C57BL/6J mice (*n* = 10 per group) exposed to low (blue), medium (green), and high (red) doses of PS-MPs, as compared to control mice (gray). Both young and old mice exposed to PS-MPs showed marked increases in (**A**–**D**) distance traveled, (**E**–**H**) rearing activity, and (**I**–**L**) duration in center. Significances were determined by unpaired *t*-test, one-way ANOVA (**B**,**D**,**F**,**H**,**J**,**L**), or two-way ANOVA with post hoc analysis (**A**,**C**,**E**,**G**,**I**,**K**) and * *p* < 0.05, ** *p* < 0.01, *** *p* < 0.001, **** *p* < 0.0001 and α *p* < 0.10 as trending.

At the end of the 3-week-long exposure, behavioral testing began. During the open-field test, mice were allowed to explore a low-lit chamber for 90 min with spontaneous movements monitored in the x-, y-, and z-directions. Several parameters to measure behavioral performance were recorded, including distance traveled, rearing activity, and duration in the center. Surprisingly, we found that acute exposure to PS-MPs induced an increase in distance traveled, which was more pronounced in older animals (Figure 3A–D). Similarly, both young and old PS-MP-exposed mice reared significantly more in the open-field, as compared to age-matched controls (Figure 3E–H). Young PS-MP-exposed mice did not spend more time in the center of the chamber overall (Figure 3J), but both low- and high-dose groups spent more time in the center when analyzed as a function of time (Figure 3I). Low- and medium-dose older animals also showed an increased duration in the center (Figure 3K,L).

**Figure 4:**
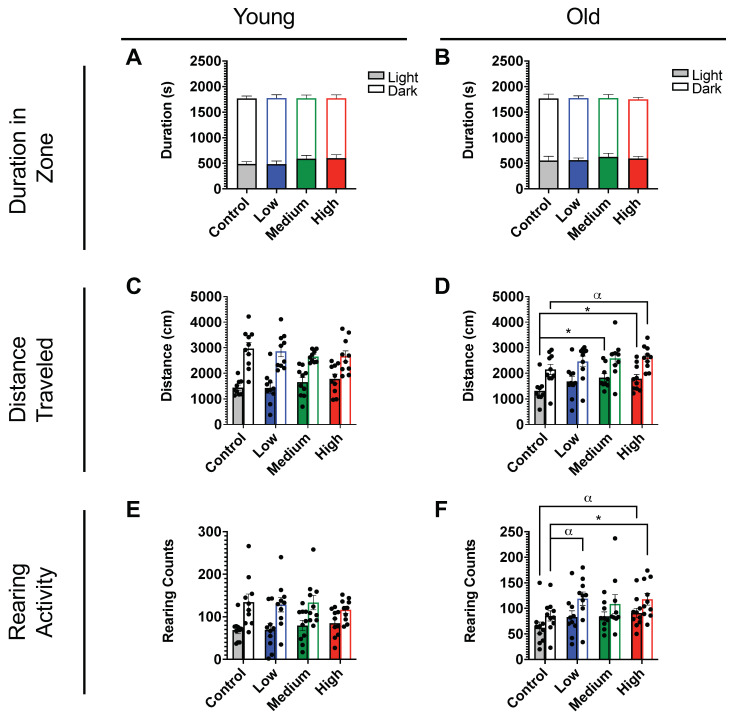
Effects of exposure to PS-MPs on light–dark preference in young and old mice. Exploratory behavior during the light-dark (solid and transparent bars, respectively) preference assay of 4- and 21-month-old female C57BL/6J mice (*n* = 10 per group) exposed to low (blue), medium (green), and high (red) doses of PS-MPs, as compared to control mice (gray). Both young and old mice exposed to PS-MPs showed striking increases in (**C**,**D**) distance traveled and (**E**,**F**) rearing activity. (**A**,**B**) No alterations were found for duration in light/dark zone. Significances were determined by unpaired *t*-test with * *p* < 0.05, and α *p* < 0.10 as trending.

In the light/dark preference test, mice were again placed in a chamber, now divided into light and dark zones, with movements monitored in the x-, y-, and z- directions. Parameters recorded for this assay included duration in zone, distance traveled, and rearing activity. PS-MP-exposure did not affect the duration spent in light and dark zones in either age group (Figure 4A,B). This assay did, however, confirm the increased distance traveled (Figure 4C,D) and rearing activity (Figure 4E,F) revealed in the open-field test (Figure 3A–H). These findings are again more pronounced in older animals.

### 2.3. Bioaccumulation of MPs

**Figure 5:**
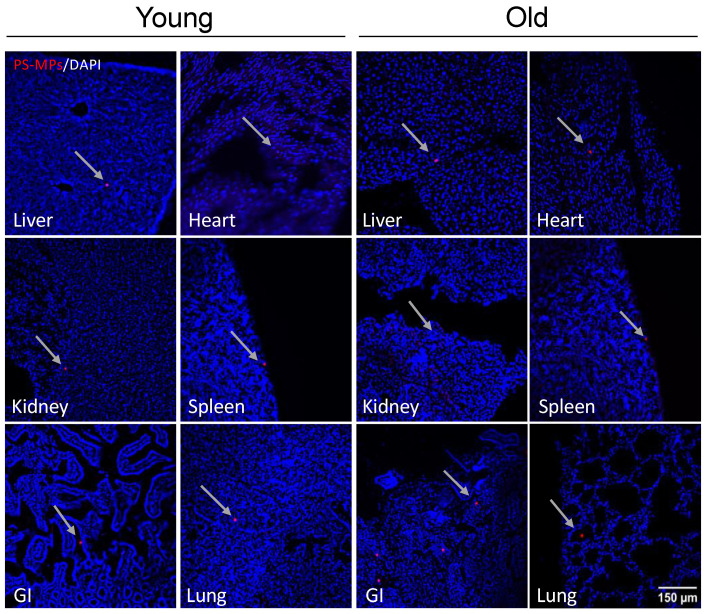
Accumulation of PS-MPs in peripheral tissues from young and old mice. Representative images of liver, kidney, gastrointestinal tract (GI), lung, spleen, and heart from young (4-month-old) and old (21-month-old) C57BL/6J mice showed presence of red fluorescent PS-MPs (red, indicted by arrows) in DAPI (blue)-stained tissues after acute (3 weeks) exposure to MPs. Scale bar = 150 μm.

**Figure 6:**
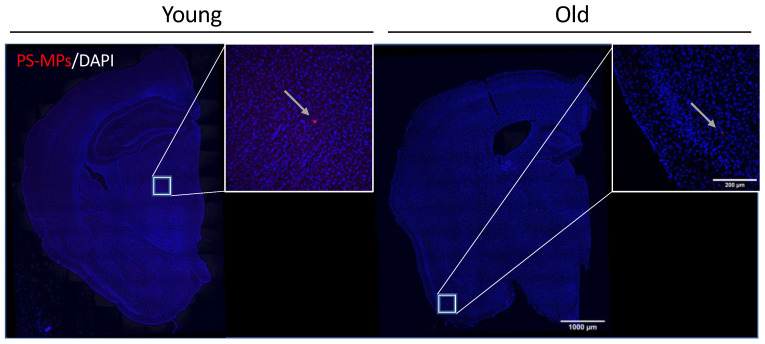
Accumulation of PS-MPs in brains from young and old mice. Representative images from young (4-month-old) and old (21-month-old) C57BL/6J mice showed presence of red fluorescent PS-MPs (red, indicated by arrows) in DAPI (blue)-stained brain tissue after acute (3 weeks) exposure to MPs. Scale bar = 1000 μm, 200 μm.

To determine if MPs in drinking water are absorbed and are able to translocate and accumulate in tissues after exposure, samples of liver, kidney, gastrointestinal tract, lung, spleen, heart, and brain tissues from both young and old exposed mice were cryosectioned and counterstained with DAPI. Unexpectedly, we detected PS-MPs within intracellular compartments of every tissue examined (Figure 5 and Figure 6). We also observed PS-MPs in urine and fecal matter.

### 2.4. Assessing Impact of MPs on Immune Markers

**Figure 7:**
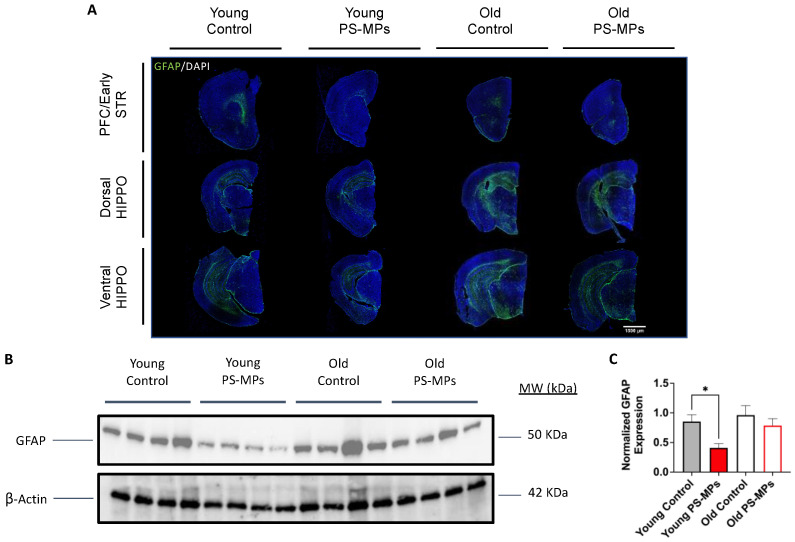
PS-MPs reduced GFAP expression in brains from young and old mice. (**A**) Representative images of GFAP (green)/DAPI (blue) fluorescent immunohistochemistry showed decreased expression throughout the brain including prefrontal cortex (PFC)/early striatum (STR) and dorsal/ventral hippocampus (HIPPO). (**B**,**C**) Decreased GFAP expression was confirmed using Western blot, with quantifications shown. Significances were determined by unpaired *t*-test with * *p* < 0.05. Scale bar = 1500 μm.

Upon observing entry of PS-MPs into the brain, we performed fluorescent immunohistochemistry of GFAP (glial fibrillary acidic protein), a marker of glial cells, which include activated astrocytes. We found decreased GFAP expression in brains from both young and old mice exposed to PS-MPs, as compared to age-matched controls (Figure 7A). A Western blot of brain lysates confirmed decreased GFAP expression (Figure 7B), and quantification revealed that this reduction was significant in young mice exposed to PS-MPs, as compared with age-matched controls (Figure 7C).

**Figure 8:**
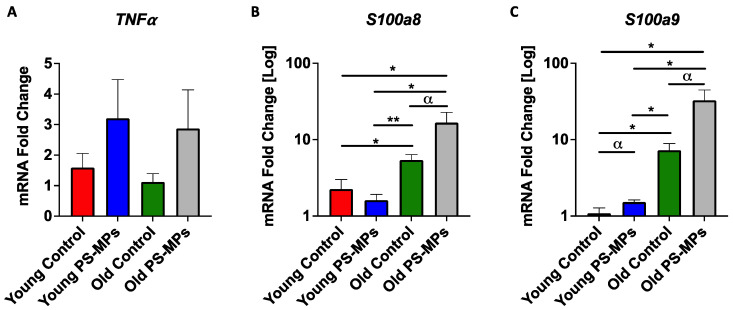
PS-MPs altered mRNA expression of inflammatory cytokine *TNF-alpha* and inflammatory-mediators *S100a8* and *S100a9* in liver tissue. (**A**) qPCR analysis showed approximately 2-fold increases in mRNA expression of *TNF-alpha* in both young and old PS-MP-exposed mice, as compared to age-matched controls. (**B**,**C**) Further analyses also showed significant increases in *S100a8* and *S100a9* mRNA expression in old mice exposed to PS-MPs, as compared to controls. Significances were determined by unpaired *t*-test with α *p* < 0.10, * *p* < 0.05, and ** *p* < 0.01.

Alterations of immune markers in liver tissues from PS-MP-exposed mice were also examined. qPCR analysis revealed a ~2-fold increase in mRNA expression of inflammatory cytokine *TNF-α* (tumor necrosis factor) in liver from young and old mice exposed to PS-MPs (Figure 8A). Calcium-binding proteins S100a8 and S100a9 (calgranulins), which mediate inflammatory responses, were also assessed. Young PS-MP-exposed mice showed little change in *S100a8* and moderate increases in *S100a9* mRNA expression. Old mice, however, exhibited higher levels of both calgranulins, as compared to young controls, and a ~3-fold increase in *S100a8* and a ~4.5-fold increase in *S100a9* mRNA expression in old mice exposed to PS-MPs, as compared to age-matched controls (Figure 8B,C).

## 3. Discussion

As global plastic production continues to rapidly grow, leading to the ubiquitous presence of microplastics, we set out to understand the potential harmful impacts of MPs in mammalian systems with a particular focus on age as a potential co-factor in adverse exposure outcomes. To first establish in vitro toxicity of 0.1 and 2 μm PS-MPs, U-2 OS cells were exposed at concentrations ranging from 0.01 to 1000 μg/mL for exposure times of 24, 48, and 72 h. Following these exposures, cell viability was assessed via an MTT assay (Figure 1A). Data collected from this assay showed that for both sizes of PS-MPs, cell viability was significantly reduced, especially as concentration and exposure time increased. Thus, suggesting that PS-MPs in this size range exhibit cytotoxicity. Additionally, PS-MPs were found to enter cells within 24 h of exposure and accumulated perinuclearly (Figure 1B).

With this in mind, an in vivo study was designed to determine the effects of these MPs in a rodent model (Figure 2A). Following a 3-week exposure to PS-MPs, C57BL/6J mice were tested in a series of behavioral assays including the open-field and light–dark preference tests. Both assays showed significant changes in parameters such as distance traveled, rearing activity, and duration in the center between the control and the exposed groups for both old and young mice (Figure 3 and Figure 4). Overall, these changes seemed to be more pronounced in older animals, which may be due to age-related dysfunction exasperating the effects of the PS-MPs on behavioral performance (Figure 3D,H,L and Figure 4D,F). The behavioral changes exhibited by the young mice, however, suggest that even without increased age as a co-variable, PS-MPs can induce altered behavior in rodents after just 3 weeks of exposure (Figure 3B,F,J and Figure 4C,E).

To understand the physiological systems that may be contributing to these changes in behavior, we began by sectioning several major tissues including the brain, liver, kidney, gastrointestinal tract, heart, spleen, and lungs to determine where these MPs may be accumulating. Surprisingly, we detected the presence of PS-MPs in every tissue examined (Figure 5 and Figure 6), as well as in urine and feces. Given that in this study the MPs were delivered orally via drinking water, detection in tissues such as the gastrointestinal tract (Figure 5), which is a major part of the digestive system, or in the liver and kidneys (Figure 5), which contribute to the detoxification of xenobiotics [35,36], was always probable. The detection of MPs in tissues such as the heart and lungs (Figure 5), however, suggests that the PS-MPs are going beyond the digestive system and likely undergoing systemic circulation. This is further supported by the detection of MPs in urine and in the brain (Figure 6), which additionally demonstrates that the PS-MPs can pass the blood–brain barrier (BBB).

Given the ability of MPs to pass the BBB, an immediate concern was the potential for these xenobiotics to trigger neuroinflammation. GFAP (glial fibrillary acidic protein), a major intermediate filament protein found in mature astrocytes that is involved in many cell processes such as autophagy, neurotransmitter uptake, and astrocyte development [37], can be used to measure the expression of activated astrocytes and is a commonly used marker in neuroinflammatory studies [38]. Astrocytes are typically activated in response to neural stress or injury [39], and because of this, an increase in GFAP expression is often associated with an increase in neuroinflammation. Fluorescent immunohistochemical staining for GFAP in PS-MP-exposed mouse brain, however, showed a slight decrease in expression for older animals and a more pronounced decrease in young mice exposed to PS-MPs, as compared to age-matched controls (Figure 7A). These results were confirmed with a Western blot analysis (Figure 7B,C). Although these results are not typical of an inflammatory response, they are consistent with previous studies that suggest that GFAP expression might decrease in early stages of certain diseases, such as Alzheimer’s disease (AD) [40,41], or in younger patients with disorders such as Major Depressive Disorder (MDD) [42]. These studies indicate that early pathology/early onset of disease may be characterized by astrocyte atrophy (as opposed to astrocyte hypertrophy later on), which may result in decreased GFAP expression. Although these mechanisms are still not well understood, our results suggest that exposure to PS-MPs results in a comparable age-dependent pattern.

Similar to our findings, results from Lee and colleagues [43] found that following either a 4- or 8-week exposure to 2 µm carboxyl-modified PS-MPs via an oral gavage, 6-week-old C57BL/6J mice exhibited accumulation of MPs in both liver and brain tissues, alterations in cognitive behavior, and modifications in immune markers in the brain. However, Lee et al. did not find any alterations in the open-field test in mice exposed to PS-MPs, in contrast to our study. This could be due to several differences between the setup of the two studies, such as age of the animals, method and length of delivery of MPs, PS-MPs having different surface chemistries, as well as Lee’s study only using one size of MPs. Despite some discrepancies between the studies, it is evident that PS-MPs can travel to and exert detrimental effects on the brain after absorption. Further studies are needed to dissect the underlining molecular mechanisms of such an effect. One possibility proposed by Lee et al. is that neurotoxic effects of PS-MPs may depend on the vagal-pathway-dependent gut–brain axis; however, other mechanisms including the impairment of blood detoxification pathways in the liver cannot be excluded. Several studies have indeed suggested that the ability of a toxin to reach the brain may be in part due to liver dysfunction, since the liver is a major site of blood detoxification. If the liver is unable to properly function, this can lead to toxin build-up in the blood [44], which may ultimately reach the brain. Similarly, hepatic failure or injury may result in increased BBB permeability [45,46]. Thus, we investigated whether PS-MPs induced an inflammatory response in the liver from young and old mice in our study and found an approximately two-fold increase in the mRNA expression of inflammatory cytokine *TNF-α* in both young and old PS-MP-exposed mice, as compared to controls (Figure 8A). Additionally, in older PS-MP-exposed mice, there was a 3-fold increase in *S100a8* mRNA expression and a 4.5-fold increase in *S100a9* mRNA expression (Figure 8B,C). S100a8 and S100a9 are Ca^2+^-binding proteins that help mediate inflammatory responses. Increased expression of these genes indicate inflammation of the liver, which may play a role in allowing the MPs to enter the bloodstream and ultimately reach the brain and other major organs.

Overall, since human exposure to MPs is inevitable due to their persistence and pervasiveness in the environment, it is essential to better understand their toxicity to limit their impact on human health. In the present study, we have shown that 0.1 and 2 μm PS-MPs can reduce cell viability, translocate throughout the body, accumulate in tissues including brain tissue, markedly modify behavior in C57BL/6J mice after only 3 weeks of exposure, and significantly alter immune markers in both the liver and the brain. Additionally, the effects of exposure seem to be age-dependent. Research into the effects of exposure to MPs in mammals is still a very broad field with many variables worth pursuing. In this study, we chose to focus specifically on the effects of exposure to pristine polystyrene microplastics (PS-MPs) via drinking water in female C57BL/6J mice. There are still many questions that remain, including but not limited to how sex, MP delivery method, length of exposure to MPs, and MP surface chemistry impact exposure outcome. While these variations were not explored in this study, future work should examine these factors in order to understand the mechanisms by which MPs exert these effects and how these mechanisms are altered with age.

## 4. Materials and Methods

### 4.1. Microplastic Particles

Dyed red aqueous pristine fluorescent polystyrene particles (PS-MPs; Thermo Scientific, Fremont, CA, USA) were obtained at diameters of 0.1 and 2 μm. MPs in this size range were selected because 0.1 μm represents the smallest end of the microplastics spectrum, bordering on nanoplastics, and 2 μm nears the upper limit of what may enter into cells [47,48]. MPs within this size range have recently been detected in human breastmilk [34], placentas [49], lungs [31], and on human hands and hair [50]. Prior to use, PS-MPs were centrifuged (Eppendorf 5424R, Hamburg, Germany) at 21,000× *g* at 4 °C for 1 h 45 min, and the supernatant was discarded. The PS-MPs were resuspended using sterile distilled water. This process was repeated 3 times to remove trace amounts of DMSO and sodium azide.

### 4.2. MTT Assay

Cell viability was assessed in vitro using an MTT assay. U-2 OS cells were seeded (Gibco, Waltham, MA, USA) in 24-well plates (Celltreat, Pepperell, MA, USA) and allowed 24 h to attach. Following this, cells were exposed to 0.01, 0.1, 1, 10, 100, and 1000 μg/mL concentrations of 0.1 and 2 μm PS-MPs for exposure times of 24, 48, and 72 h. At the end of exposure, 200 μL of MTT solution (5 mg/mL 3-(4,5-Dimethylthiazol-2-yl)-2,5-Diphenyltetrazolium Bromide (Alfa Aesar, Ward Hill, MA, USA)) in 0.01 M sterile PBS (Corning, Corning, NY, USA) diluted 1:6 in Dulbecco’s Modified Eagle Medium (DMEM; Gibco, Waltham, MA, USA) was added to each treated well. Negative controls received 200 μL of MTT solution, and positive controls received 180 μL of MTT solution and 20 μL of DMSO (MilliporeSigma, Burlington, MA, USA). Following this, plates were incubated at 37 °C for 2.5 h. After completion of the MTT reaction, cells were washed with 0.01 M PBS, and 200 μL of DMSO was added to each well. The plates were placed back in the incubator for 15 min before being placed on an orbital shaker at RT for 15 min. A total of 150 μL from each well was transferred to a 96-well plate, and absorbance was measured at 485 nm.

### 4.3. Visualization of Red Fluorescent MPs in Cells

U-2 OS cells were cultured and plated in a 6-well plate (Celltreat, Pepperell, MA, USA). Once adherent, cells were treated with 5 μg/mL of 0.1 μm PS-MPs or 20 μg/mL of 2 μm PS-MPs for 24 h. After exposure, the cells were washed with sterile PBS (Gibco, Waltham, MA, USA), detached, centrifuged in DMEM (Gibco, Waltham, MA, USA) at 1000× *g* at 4 °C for 5 min to remove excess PS-MPs, and re-plated on 13 mm coverslips in a 6-well plate. After 24 h, the cells were washed 3 times with PBS, fixed for 10 min with 4% formaldehyde at RT in PBS, permeabilized with 0.1% Triton X-100 (Sigma, St. Louis, MO, USA) in PBS at RT for 15 min, stained with Hoescht (1:2000, H1399, Invitrogen, Waltham, MA, USA) and Phalloidin (1:500, PF7501, EPM Scientific, New York, NY, USA) for 5 min at RT, washed 3 times in PBS, and mounted onto glass slides with an aqueous mounting medium. Fluorescence imaging (Leica THUNDER DMi8 3D Fluorescence Imaging System, Leica Biosystems, Wetzlar, Germany and LAS X 3D Analysis Software v. 2018.7.3, Leica Biosystems, Wetzlar, Germany) was used to identify the dyed red fluorescent polystyrene particles using a TRITC (550 nm) filter.

### 4.4. Animals and Exposure

Both young (4-month-old, *n* = 40) and old (21-month-old, *n* = 40) female C57BL/6J mice were obtained from the National Institute of Aging (NIA) aged rodent colony (Charles River Laboratories, Kingston, NY or Raleigh, NC, USA). Female mice were selected based on their ability to be re-housed after the mice were delivered and acclimated in our animal facility in order to minimize weight discrepancies between treatment groups. All mice were acclimated for at least 2 weeks in our animal facility prior to testing. Within each age cohort, four exposure groups (*n* = 10 per group) were established to receive 1:1 ratios of 0.1 and 2 μm PS-MPs via drinking water: normal drinking water (control group), 0.0025 mg/mL (low-dose group), 0.025 mg/mL (medium-dose group), and 0.125 mg/mL (high-dose group). Drinking water was selected as the delivery vehicle over oral gavage in order to allow for continuous exposure as opposed to timed bursts, as well as to minimize external stress that may impact behavior performance. Water consumption and body weights were monitored throughout exposure to ensure comparable exposure between groups (Figure S1). Water bottles were mixed every 10–12 h; mice were exposed for three weeks, during which time the drinking water was replaced as needed, i.e., every ~10–12 days. Exposure dosages were selected according to previous studies [18]. All mice received a standard diet (Teklad Global Soy Protein-Free [Irradiated] type 2920X, Envigo, Indianapolis, IN, USA) and water ad libitum; they were group-housed based on how the mice were received from the source institution with up to 5 mice per ventilated cage with access to a small house and tissues for nesting. The mice were kept on a 12:12 light: dark cycle at 22 °C ± 1 and 30–70% humidity. Adequate measures were taken to minimize animal pain and discomfort. The investigation was conducted in accordance with the ethical standards and according to the Declaration of Helsinki and national and international guidelines and has been approved by the authors’ institutional review board.

### 4.5. Behavior Experiments

The mice were acclimated in their home cage for 1 h in the testing room and were kept at 22 °C ± 1, 30–70% humidity, and ~100 lux prior to testing. The testing room was in a neutral, quiet environment, and mice were tested between 9:00 and 17:00 (light phase) by the same researcher, with care taken to stagger the testing of mice from the different exposure and age cohorts. The mice were transported to and from the apparatus in a non-transparent plastic container cleaned with 70% ethanol after each use.

### 4.6. Open-Field Test (OF)

A multi-cage infrared-sensitive motion detection system (Fusion v6.5 SuperFlex, Omnitech Electronics, Columbus, OH, USA) was used to assess exploratory behavior and spontaneous locomotion. During this assay, the mice were placed in darkened transparent locomotor chambers (40 × 40 × 30 cm) equipped with a grid of infrared beams at floor level and 7.5 cm above floor level for 90 min while their movements were monitored in 5 min intervals in the x-, y-, and z-planes. All horizontal and vertical movements were recorded, and data were analyzed using the Fusion v6.5 software system. Locomotor boxes were cleaned with 70% ethanol after each test period.

### 4.7. Light–Dark Preference Test (LD)

To assess exploratory and anxiety-related behaviors, all mice were placed in locomotor chambers (40 × 40 × 30 cm) divided into light and dark zones that were equipped with a grid of infrared beams at floor level and 7.5 cm above floor level. All mice were tested for 30 min with their movements being monitored in the x-, y-, and z-planes using an infrared-sensitive activity-monitoring cage system (Fusion v6.5 SuperFlex, Omnitech Electronics, Columbus, OH, USA). All movements were recorded, and data were analyzed using the Fusion v6.5 software system. Locomotor boxes and inserts were cleaned with 70% ethanol after each test period.

### 4.8. Tissue Preparation

All mice were anesthetized with sodium pentobarbital (200 mg/kg) intraperitoneally and were killed by cervical dislocation; brain, lungs, heart, liver, kidneys, gastrointestinal tract (GI), spleen, and gastrocnemius muscle tissues were either post-fixed in 10% formalin (Epredia, Portsmouth, NH, USA) for 24 h at 4 °C, followed by 30% sucrose (*w*/*v*) in 1X PBS, or rapidly frozen on dry ice, embedded (Tissue-Plus^®^ OCT compound, Fisher Scientific, Waltham, MA, USA), and stored at −80 °C.

### 4.9. Visualization of Red Fluorescent MPs in Tissues

For detection of MPs, representative post-fixed brain, lung, heart, liver, kidney, GI, and spleen samples from control and high-dose groups from both young and old cohorts were examined. Frozen embedded tissues were sectioned at 20 μm using a cryostat (Leica BioSystems, CM1950, Wetzlar, Germany) taken at −21 °C and collected onto glass slides (VWR Colorfrost^®^ Plus, Radnor, PA, USA). The sections were allowed to dry at 30 °C for 5–10 min before being circled using a wax pen. The sections were washed for 5 min in TBS, permeabilized with 0.3% Triton X-100 (Sigma, St. Louis, MO, USA) in TBS for 30 min, and stained with DAPI (1:5000, 5.08741.0001, MilliporeSigma, Burlington, MA, USA) for 10 min. Sections were washed again in TBS for 5 min and coverslipped (VWR, Radnor, PA, USA) with aqueous anti-fading mounting medium (20 mM Tris pH 8.0, 0.5% N-propyl gallate, 50% glycerol). Fluorescence imaging (Leica THUNDER DMi8 3D Fluorescence Imaging System, Leica Biosystems, Wetzlar, Germany and LAS X 3D Analysis Software v. 2018.7.3, Leica Biosystems, Wetzlar, Germany) was used to identify the dyed red fluorescent polystyrene particles using a TRITC (550 nm) filter.

### 4.10. Fluorescent Immunohistochemistry (IHC)

For determining GFAP expression, frozen and embedded post-fixed brain samples from control and high-dose groups from both the young and old cohorts were cryosectioned (Leica BioSystems, CM1950, Wetzlar, Germany) at 30 μm taken at −21 °C; free-floating sections were collected into 12-well plate netwells (3477, Corning, Corning, NY, USA) filled with PBS using previously reported methodology [51]. Briefly, sections were blocked in TBS with 3% horse serum and 0.3% Triton X-100 at RT for 30 min on an orbital shaker and incubated overnight at 4 °C on a rotator with rabbit anti-GFAP primary antibody (1:2000, PA1-10019, Invitrogen, Waltham, MA, USA) in TBS with 1% horse serum and 0.3% Triton X-100. Sections were incubated the following day with donkey anti-rabbit Alexa 488 (1:500, A21202, Invitrogen, Waltham, MA, USA) secondary antibody in TBS with 1% horse serum and 0.3% Triton X-100 at RT for 2 h on an orbital shaker, while being protected from light. This was followed by DAPI (1:5000, 5.08741.0001, MilliporeSigma, Burlington, MA, USA) staining. Sections were mounted onto slides (VWR Colorfrost^®^ Plus, Radnor, PA, USA), coverslipped with aqueous mounting medium, and dried at RT for 15–20 min protected from light. Fluorescence imaging (Leica THUNDER DMi8 3D Fluorescence Imaging System, Leica Biosystems, Wetzlar, Germany and LAS X 3D Analysis Software v. 2018.7.3, Leica Biosystems, Wetzlar, Germany) was used to evaluate GFAP expression.

### 4.11. Western Blot (WB)

Brain samples from young and old control and high-dose cohorts were lysed in RIPA buffer (50 mM Tris-HCl pH 7.4, 150 mM NaCl, 0.5% deoxycholic acid, 0.1% sodium dodecyl sulfate, 2 mM EDTA, 1% Triton X-100) containing a proteinase (1:100, 78,438, Halt, Thermo Scientific, Fremont, CA, USA) and phosphatase (1:100, P0044, Sigma Aldrich) inhibitor cocktail. Samples were incubated on ice for 30 min and then sonicated (QSonica, Newtown, CT, USA) for 3 min (30–30 pulse) at 4 °C with 30% amplitude and centrifuged at 10,000× *g* for 10 min. Protein concentration was determined using a BCA protein assay kit (23,225, Thermo Scientific, Fremont, CA, USA) according to the manufacturer’s instructions. Lysates were mixed with loading buffer (1610747, Bio-Rad, Hercules, CA, USA) plus 100 mM DTT and incubated at 98 °C for 10 min. Samples were fractionated in an SDS-PAGE precast 8–16% gradient gel (5671104, Bio-Rad, Hercules, CA, USA) and blotted on a 0.2 μm nitrocellulose membrane (1620112, Bio-Rad, Hercules, CA, USA) using transfer buffer (25 mM Tris-HCl pH 8.3, 190 mM glycine 20% methanol). The membrane was blocked (0.1% TBS-Tween with 5% skim milk) and probed with rabbit anti-GFAP primary (1:10,000, PA1-10019, Invitrogen, Waltham, MA, USA) and goat anti-rabbit-HRP secondary antibodies in 0.1% TBS-Tween (1:3000, 1706515, Bio-Rad, Hercules, CA, USA); then, immunocomplexes were detected by chemiluminescence (Clarity ECL, Bio-Rad, Hercules, CA, USA) and visualized (ChemiDoc, BioRad, Hercules, CA, USA). Images were processed and quantified using appropriate software (FIJI v2.1.0/1.53c, Madison, WI, USA) [52].

### 4.12. Quantitative Real-Time PCR (qPCR)

Samples of liver tissue (30–50 mg) from young and old control and high-dose groups were lysed, and RNA was extracted and purified (Zymo Direct-zol RNA MiniPrep Plus Kit R2070, Zymo Research, Irvine, CA, USA) according to the manufacturer’s instructions. RNA concentration was determined using a spectrophotometer (NanoDrop, ND-2000, Thermo Scientific, Fremont, CA, USA), and reverse transcription was run (Lunascript^®^ RT Supermix Kit E3010, New England BioLabs Inc., Ipswich, MA, USA) according to the manufacturer’s protocol. Once the cDNA was synthesized, qPCR reactions were run using a SYBR green-based master mix (Luna^®^ qPCR Mastermix M3003, New England BioLabs Inc.) according to the manufacturer’s instructions using appropriate instrumentation (Viia7 Real-Time PCR System, Applied Biosystems, Waltham, MA, USA) and the following conditions: 95 °C for 15 s, 95 °C for 15 s with optimal annealing temperature for each gene of interest (varied by gene) for 30 s × 40, and melt curve of 65–90 °C. The results were analyzed using the appropriate software (GraphPad Prism v. 9, San Diego, CA, USA). The primer sequences were as follows: *TNFα*_For_: GGTGCCTATGTCTCAGCCTCTT, *TNFα*_Rev_: GCCATAGAACTGATGAGAGGGAG; *s100a8*_For_: CCTTTGTCAGCTCCGTCTTCA, *s100a8*_Rev_: TCCAGTTCAGACGGCATTGT; *s100a9*_For_: AATGGTGGAAGCACAGTTGG, *s100a9*_Rev_: CTGGTTTGTGTCCAGGTCCTC. 

### 4.13. Microscopy

Fluorescence imaging was used to evaluate and document MPs and the immunolabeling results (Leica THUNDER DMi8 3D Fluorescence Imaging System, Leica Biosystems, Wetzlar, Germany, and LAS X 3D Analysis Software v. 2018.7.3, Leica Biosystems, Wetzlar, Germany). Images were processed using the appropriate software (FIJI v2.1.0/1.53c, Madison, WI, USA) [52].

### 4.14. Statistical Analysis

Data are presented to three significant digits as mean values (Ms) with SEM and are indicated in the figure legend together with sample size (N). Statistical analyses, unpaired t-tests, one-way ANOVAs, or two-way ANOVAs with Tukey posthoc multiple comparisons were performed with an α level of 0.05 using the appropriate software (GraphPad Prism v. 9, San Diego, CA, USA). Significances are denoted in figures with * *p* < 0.05, ** *p* < 0.01, *** *p* < 0.001, **** *p* < 0.0001, and trending with α *p* < 0.10.

## Data Availability

Data generated from this study are available upon request.

## Supplementary Materials

The following supporting information can be downloaded at: https://www.mdpi.com/article/10.3390/ijms241512308/s1.
