# Supplementary material for: Acute Exposure to Microplastics Induced Changes in Behavior and Inflammation in Young and Old Mice
Source: Int J Mol Sci. 2023 Aug 1;24(15):12308. doi: 10.3390/ijms241512308 (PMC10418951; doi:10.3390/ijms241512308)
Supplement: Supplementary file 1 [file ijms-24-12308-s001.zip › ijms-2527708-supplementary.pdf]

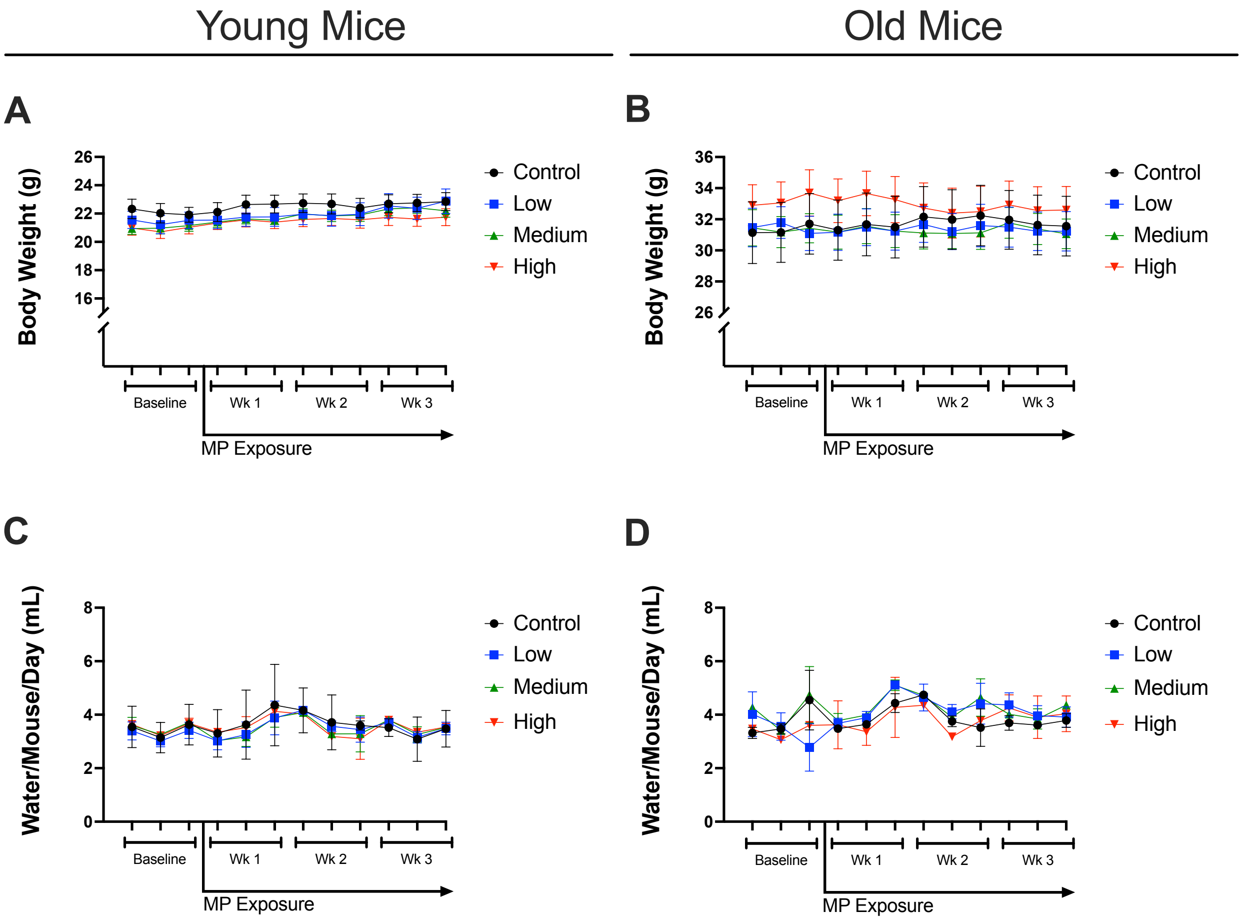

Supplementary Figure S1. Body weights and water consumption. (A, B) Gross body weights of 4- and 21-month old female C57BL/6J mice (N=10 per group) exposed to low (blue), medium (green), and high (red) doses of PS-MPs, as compared to control mice (gray). Body weights were measured 3 times per week for one week prior to PS-MPs exposure and then weekly during the 3 week exposure period. No significant differences in body weight were observed for any exposed group, as compared to controls in young (2-way RM ANOVA: low: p=0.5795, medium: p=0.3559, high: p=0.2168) and old mice (2-way RM ANOVA: low: p=0.8599, medium: p=0.8281, high: p=0.6612). **(C, D)** Average water consumption of 4- and 21-month old female C57BL/6J mice (N=10 per group) exposed to low (blue), medium (green), and high (red) doses of PS-MPs, as compared to control mice (gray). Water consumption was measured 3 times per week before and during PS-MPs exposure. Both young and old PS-MPs-exposed mice showed no significant differences in water consumption, as compared to controls (2-way RM ANOVA$_{YOUNG}$: low: p=0.8947, medium: p=0.8614, high: p=0.9201; 2-way RM ANOVA$_{OLD}$: low: p=0.3400, medium: p=0.2843, high: p=0.9845).
